# Eating, sleeping and moving recommendations in clinical practice guidelines for paediatric depression: umbrella review

**DOI:** 10.1192/bjo.2021.1020

**Published:** 2021-10-05

**Authors:** Susan C. Campisi, Karolin R. Krause, Benjamin W. C. Chan, Darren B. Courtney, Kathryn Bennett, Daphne J. Korczak, Peter Szatmari

**Affiliations:** Department of Psychiatry, Hospital for Sick Children, Canada; Cundill Centre for Child and Youth Depression, Centre for Addiction and Mental Health, Canada; Evidence-Based Practice Unit, University College London, UK; and Evidence-Based Practice Unit, Anna Freud National Centre for Children and Families, UK; Independent practitioner, Canada; Cundill Centre for Child and Youth Depression, Centre for Addiction and Mental Health, Canada; and Department of Psychiatry, University of Toronto, Canada; Department of Health Research Methods, Evidence and Impact, McMaster University, Canada; Department of Psychiatry, Hospital for Sick Children, Canada; and Department of Psychiatry, University of Toronto, Canada; Department of Psychiatry, Hospital for Sick Children, Canada; Cundill Centre for Child and Youth Depression, Centre for Addiction and Mental Health, Canada; and Department of Psychiatry, University of Toronto, Canada

**Keywords:** Depressive, adolescent clinical practice guidelines, sleep, exercise, diet

## Abstract

**Background:**

Current first-line treatments for paediatric depression demonstrate mild-to-moderate effectiveness. This has spurred a growing body of literature on lifestyle recommendations pertaining to nutrition, sleep and exercise for treating paediatric depression.

**Aims:**

Paediatric depression clinical practice guidelines (CPGs) were reviewed for quality and to catalogue recommendations on nutrition, sleep and exercise made by higher-quality CPGs.

**Method:**

Searches were conducted in Medline, EMBASE, PsycINFO, Web of Science and CINAHL, and grey literature CPGs databases for relevant CPGs. Eligible CPGs with a minimum or high-quality level, as determined by the Appraisal of Guidelines for Research and Evaluation, Second Edition instrument, were included if they were (a) paediatric; (b) CPGs, practice parameter or consensus or expert committee recommendations; (c) for depression; (d) the latest version and (e) lifestyle recommendations for nutrition, sleep or exercise. Key information extracted included author(s), language, year of publication, country, the institutional body issuing the CPG, target disorder, age group, lifestyle recommendation and the methods used to determine CPG lifestyle recommendations.

**Results:**

Ten paediatric CPGs for depression with a minimum or high-quality level contained recommendations on nutrition, sleep or exercise. Lifestyle recommendations were predominately qualitative, with quantitative details only outlined in two CPGs for exercise. Most recommendations were brief general statements, with 50% lacking supporting evidence from the literature.

**Conclusions:**

Interest in lifestyle interventions for treatment in child and youth depression is growing. However, current CPG lifestyle recommendations for nutrition, sleep or exercise are based on expert opinion rather than clinical trials.

Adolescents (ages 10–19 years) represent 1.2 billion of the world's population, which is the largest generation in this age stratum in human history.^1^ Annually, it is estimated that up to 20% of adolescents will experience a mental health disorder and 16% of the global burden of disease and injury among 10- to 19-year-olds are mental health conditions.^2^ According to the World Health Organization, depression is one of the most common causes of disability in adolescence.^3^ The development of a mental health disorder like depression before 20 years of age has been associated with an increased risk for other mental health conditions in adulthood,^4^ including substance dependence,^5^ suicide,^6^ cardiovascular diseases^7^ and obesity.^8^ Depression during adolescence can undermine educational achievement, employment prospects and the ability to establish intimate relationships, which can have lifelong repercussions.^9^

First-line psychotherapeutic and psychopharmacological treatments have shown mild-to-moderate effectiveness for treating depression in youth. These include cognitive–behavioural therapy (CBT), interpersonal psychotherapy and antidepressant medication.^10^ Yet, many children or adolescents do not respond to first-line treatment(s), and the prevalence of treatment-resistant depression is 30–40%.^11^ Strategies to enhance treatment effectiveness include switching medication, pharmacologic and psychotherapy augmentation strategies, and innovative e-health interventions,^12^ but the search for effective alternative treatment approaches (either adjunctive or standalone) is ongoing.

During adolescence, life-shaping behaviours develop, including eating habits, sleep patterns and physical activity routines. These, in turn, can affect current and future health: emerging literature discussed in the following suggests some benefit to paediatric depression for lifestyle interventions such as improving nutrition, sleep and physical activity. Lifestyle interventions may be less resource-intensive compared with specialist psychotherapeutic interventions. For example, a relevant treatment plan might be prescribed and monitored by a primary care physician within a stepped-care model,^13^ before considering the prescription of medication or referral into specialist mental healthcare. Specialist mental healthcare may also combine several treatment components. For example, CBT might be combined with psychoeducation and parental counselling. Lifestyle interventions for these and other lifestyle aspects not explicitly covered in this study (e.g. screen time) may be additional strategies used as part of such a sequence. Lifestyle interventions may also offer an approach to addressing depression in contexts where trained psychiatrists or clinical psychologists are not widely available, and where these interventions may provide low-stigma access to mental health treatment.

## Nutrition interventions for paediatric depression

The field of nutritional psychiatry is in its infancy. A recent evidence gap map reveals a paucity of research, and especially randomised controlled trials, assessing micronutrients in the treatment of depression in children and adolescents.^14^ Among cross-sectional studies, two recent systematic reviews without meta-analyses highlight an association between unhealthy diets and depressive symptoms among children and adolescents.^15,16^ Much needs to be gleaned about the physiological impact of suboptimal nutrition on brain function, but it is generally accepted that optimal brain function requires adequate levels of both macronutrients (e.g. fat, carbohydrates and protein) and micronutrients (e.g. vitamins and minerals).^17^ Ideal typical high-quality diets, such as the Mediterranean diet, are characterised by substantial intake of whole grains, fruits/vegetables and legumes; moderate amounts of fish, seafood, dairy, poultry and healthy fats; and low intake of red meat and sweets. They are thought to be beneficial because they are high in antioxidants, polyunsaturated fat, dietary fibre, vitamins and minerals; and low in saturated fat, simple sugar and processed food.^18^

## Sleep interventions for paediatric depression

Sleep disturbance is one of the most common symptoms among adolescents with depression,^19^ and is correlated with depression severity.^20^ To date, two meta-analyses have found an association between poor sleep quality and depression in adolescents.^21,22^ Clinical interventions to restore healthy sleep routines and good sleep quality have demonstrated improvements in depressive symptoms in youth. One small feasibility trial observed medium-sized effects of improved outcomes among youth diagnosed with both depression and insomnia, compared with a CBT and sleep hygiene control condition when CBT for depression was augmented with CBT for insomnia.^23^ A randomised controlled trial comparing a sleep extension intervention (involving bedtime scheduling and sleep hygiene advice) with an attention placebo in adolescents with chronic sleep reduction also found significant improvement in depressive symptoms in the intervention group, compared with the control group (β = 0.41, s.e. 0.16, *P* = 0.01).^24^ Among this body of literature, such trials suggest that interventions are promising, although systematic reviews have not been conducted, as the evidence base for the effect of adjunctive or standalone sleep interventions remains scarce.^23^

## Exercise interventions for paediatric depression

Low physical activity and high sedentary behaviour are emerging as risk factors for depression. The largest body of evidence for paediatric lifestyle interventions exists for exercise. A recent systematic review of reviews examining physical activity for the prevention of depression demonstrated a beneficial effect of exercise on depressive symptoms across all age groups, including children and adolescents.^25^ Among children and adolescents with elevated or clinical levels of depressive symptoms, a moderate-to-large effect size for efficacy was reported in using physical activity interventions as treatment in two recent meta-analyses.^26,27^

Although the evidence base supporting lifestyle interventions for the treatment of youth depression is still comparatively scarce, there exists a growing interest in the active ingredients of treatment for depression.^28^ As adjunctive treatments, lifestyle interventions could possibly enhance the effectiveness of evidence-based psychotherapies or medication; in stepped-care^13^ models, clinicians may consider lifestyle interventions for the treatment of depressed youth, especially in primary care.

Clinical practice guidelines (CPGs) are defined as ‘systematically developed statements to assist practitioner and patient decisions about appropriate healthcare for specific clinical circumstances’.^29^ High-quality CPGs derived from the findings of rigorous systematic reviews and meta-analyses represent the highest level of evidence in evidence-based medicine. Their development involves systematic and methodologically rigorous procedures for reviewing and appraising the evidence, and deriving feasible, measurable and achievable recommendations.^29^ This umbrella review aims to investigate the extent to which nutrition, sleep and exercise interventions are part of current recommended practice for treating paediatric depression around the world; to critically examine the quality of the CPGs issuing relevant guidance; and finally, to catalogue recommendations made by high-quality CPGs.

## Method

An integral component of CPGs is a rigorous review of evidence-based methodology. To systematically review CPGs, we followed the Joanna Briggs Institute methodology for umbrella reviews,^30^ with considerations specific to CPGs.^31^

### Search strategy

We drew on an existing database of CPGs for paediatric depression that was created as part of a previous systematic search, and included CPGs published in the English language between 2005 and July 2017.^32,33^ The database was updated and expanded to include CPGs published in English, French, German and Spanish (i.e. languages spoken within the study team), up until July 2020. There was no limit on geographical location.

The search strategy (Supplementary Table 1 available at https://doi.org/10.1192/bjo.2021.1020) was developed by a research librarian. CPGs were retrieved from five bibliographic databases (Medline, EMBASE, PsycINFO, Web of Science and CINAHL), and a thorough grey literature search in relevant CPGs databases (e.g. National Guideline Clearinghouse, Guidelines International Network, Australia's Clinical Practice Guidelines Portal); websites of mental health-related organisations (e.g. American Academy of Child and Adolescent Psychiatry, Canadian Mental Health Association), governmental websites and a broad Google search.

### Screening

For the creation of the initial CPGs database, titles and abstracts were screened by a methodologist and eligible full texts were then screened in duplicate for inclusion by two methodologists.^33^ For the updated and expanded search, title and abstract screening was conducted by K.R.K., and a second reviewer (B.W.C.C.) screened 10% of records in duplicate, yielding a high interrater agreement (κ = 0.86). All full texts in the database were screened for inclusion in this umbrella review in duplicate by two reviewers (K.R.K. and S.C.C.), with a high interrater agreement (κ = 0.90). Disagreements were resolved by discussion. Title and abstract screening were conducted with the web-based systematic review software EPPI-Reviewer version 4.0 (University of London, UK, see; https://eppi.ioe.ac.uk/cms/Default.aspx?alias=eppi.ioe.ac.uk/cms/er4).^34^ The full-text screening was conducted with Covidence web-based software (Veritas Health Innovation, Melbourne, Australia; see https://www.covidence.org/).^35^

### Eligibility criteria

Records were eligible if they were relevant to the paediatric population (<19 years of age); labelled as CPGs, practice parameter or consensus or expert committee recommendations; addressed psychosocial treatment for depressive disorder; were the latest version of the CPG; and contained any reference to lifestyle recommendations for nutrition, sleep or exercise. Lifestyle recommendations regarding nutrition, sleep or exercise could be brief standalone statements or detailed recommendations. CPGs were excluded if they contained only medication algorithms or were narrative or systematic literature reviews that contained no summary statements or algorithms regarding CPG recommendations.

### Quality assessment

Eligible CPGs were assessed for quality with the Appraisal of Guidelines for Research and Evaluation, Second Edition (AGREE-II)^36^ instrument, comprising 23 items with six domains. Two pairs of reviewers (including S.C.C., K.R.K. or B.W.C.C.) appraised all English guidelines with the AGREE–II online software (https://www.agreetrust.org/agree-ii/). In cases when the overall scores differed by more than two points, all domains were reviewed, and final decisions were determined by consensus. German, French or Spanish CPGs were appraised by K.R.K. Three key domains of the AGREE-II were used to determine the quality of the CPGs: stakeholder involvement, the rigor of development and editorial independence. These three key domains have been used previously to assess the magnitude of the risk of bias in each CPG.^32^ AGREE-II scores of ≥70% indicated high quality, scores from 50 to <70% indicated minimum acceptable quality, and scores <50% indicated low quality. Only CPGs of minimum or high quality were included in this umbrella review.

### PICAR statement

For systematic reviews of CPG recommendations, the Population, Intervention, Comparator, Outcome (PICO) statement is adapted into a PICAR statement, where P stands for the target population, clinical indication(s) and condition(s); I stands for target intervention(s); C stands for eligible comparators or comparisons, and key content; A stands for attributes of eligible CPGs and R stands for recommendation characteristics (Table 1).^31^

**Table 1:** PICAR elements relevant to screening CPGs recommendations for inclusion

PICAR item	PICAR elements relevant to screening CPG recommendations for inclusion
P: Population, clinical indication(s) and condition(s)	Paediatric population (<19 years of age); depressive disorder
I: Intervention(s)	Lifestyle interventions including to nutrition, sleep or exercise
C: Comparator(s), comparison(s) and (key) content,	Only paediatric CPGs that discuss nutrition, sleep or exercise
A: Attributes of eligible CPG	The latest version of a CPG published between 2005 and 2020 Language of publication: English, French, German or Spanish Clinical focus: prevention or treatment Intended end-user: practitioners Minimum or high-quality score in the three key domains of the AGREE-II instrument
R: Recommendation characteristics	Must discuss at least one eligible lifestyle intervention. They can be presented separately in recommendation statements, evidence summaries, tables, decision algorithms or decision paths.

AGREE-II, Appraisal of Guidelines for Research and Evaluation, Second Edition; CPG, clinical practice guideline.

### Data extraction

Key information was extracted from included CPGs as follows: author(s), language, year of publication, country (i.e. where the work was published or conducted), the institutional body issuing the CPG, target disorder, age group, lifestyle recommendation and methods used to determine CPG lifestyle recommendations. Extraction forms were pilot tested. Extraction was conducted in Microsoft Excel 2021 for Windowsby one reviewer (S.C.C. or K.R.K.), and verified by the second reviewer. Narrative passages extracted from foreign-language CPGs were translated for citation in this paper by K.R.K.

## Results

**Fig. 1:**
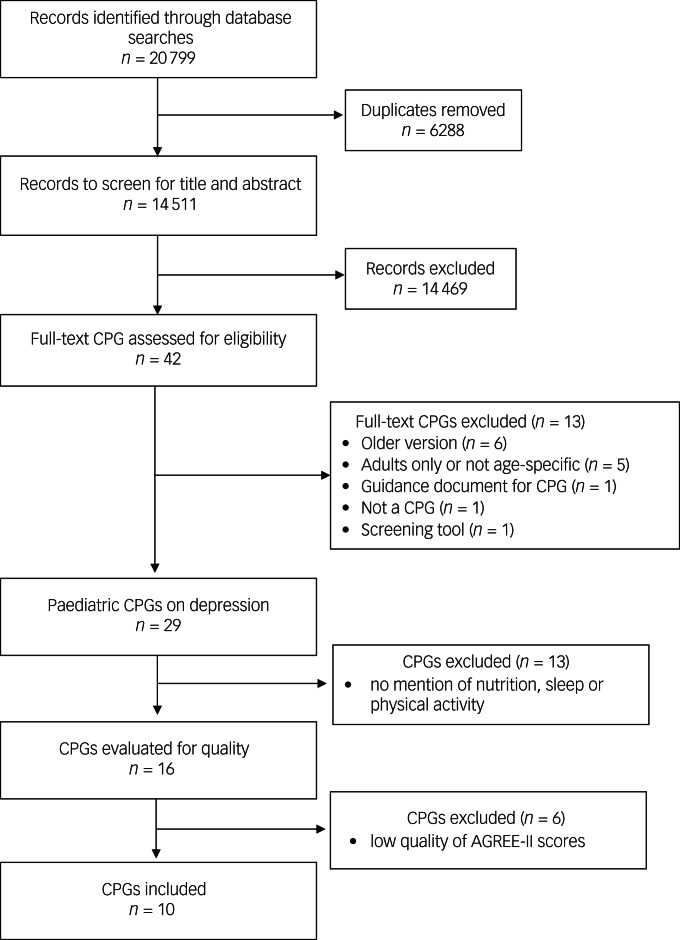
Study Preferred Reporting Items for Systematic Reviews and Meta-Analyses (PRISMA) flowchart. AGREE-II, Appraisal of Guidelines for Research and Evaluation, Second Edition; CPG, clinical practice guidelines.

After completing the two-stage record screening, 16 of the 29 paediatric CPGs for depression were found to contain nutrition, sleep or exercise recommendations. The Preferred Reporting Items for Systematic Reviews and Meta-Analyses flowchart can be found in Fig. 1. Minimum acceptable quality as per the AGREE-II assessment was observed for ten CPGs in the current umbrella review, and six CPGs were excluded because of the low quality of their AGREE-II scores (Supplementary Table 2). Among the included CPGs, six were written in English, three in Spanish and one in French. Half of the included CPGs (*n* = 5) were specific to children and adolescents, three focused only on adolescents and two on adolescents and young adults. CPGs targeted treatment for general depression (*n* = 6), major depressive disorder/major depression (*n* = 2) and depressive disorders (*n* = 1). Based on quality ratings in the three key domains of the AGREE-II appraisal, five of the included CPGs were rated to be of high quality (AGREE-II score >70), and five CPG scored in the minimally acceptable range (AGREE-II score 50 to <70) (Table 2).

**Table 2:** Quality assessment AGREE-II scores (*n* = 10)

Clinical practice guideline	Agree-II domain						Rating		
	Scope and purpose	Stakeholder involvement	Rigor of development	Clarity of presentation	Applicability	Editorial independence	Combined overall rating	Three key domains score^a^	Non-key domains score^b^
Grupo de Trabajo, 2018^38^	100	83	71	89	58	100	83	85	82
National Institute for Health and Care Excellence, 2019^37^	83	72	72	83	83	92	92	79	83
MacQueen et al, 2016^41^	89	78	70	67	60	83	75	77	72
Beyond Blue, 2011^42^	69	81	63	92	52	83	83	76	71
Cheung et al, 2018^43^	97	75	82	92	42	71	83	76	77
Driot et al, 2020^44^	67	83	44	44	29	75	50	67	47
Seo et al, 2018^45^	75	64	50	78	56	71	67	62	70
Ministerio de Salud, 2013a^46^	89	61	58	72	29	42	67	54	63
Garland et al, 2016^47^	78	53	63	56	48	42	58	53	61
Ministerio de Salud, 2013b^48^	83	61	67	67	29	25	67	51	60

AGREE-II, Appraisal of Guidelines for Research and Evaluation, Second Edition.

a. Three key domains (stakeholder involvement, rigor of development, editorial independence).

b. Non-key domains (scope and purpose, clarity of presentation, applicability); high-quality score ≥70; minimum quality score 50 to <70; low-quality score <50.

Four out of the five high-quality CPGs provided references to relevant primary evidence alongside the lifestyle recommendations, whereas four out of the five CPGs scoring in the minimally acceptable quality range did not reference evidence sources for their recommendations.

All eight recommendations related to nutrition were brief statements, suggesting ‘adequate/balanced nutrition’ or ‘healthy eating’, and were accompanied by sleep and/or exercise recommendations. Eight CPGs providing recommendations related to sleep made brief statements and supported general good sleep ‘habits’ or ‘patterns’ or ‘hygiene’. Sleep recommendations were accompanied by other lifestyle recommendations. Six of the eight CPGs providing exercise recommendations were general and included statements such as ‘benefits of regular exercise’ and ‘exercise routines’. Two CPGs provided more detailed recommendations concerning exercise and offered a quantitative component (see below) with citations from the literature. Both CPGs had a high-quality AGREE-II score in the three key domains. The first was the guideline issued by the National Institute for Health and Care Excellence (NICE) in the UK, which suggested that:

‘A child or young person with depression should be offered advice on the benefits of regular exercise and encouraged to consider following a structured and supervised exercise program of typically up to 3 sessions per week of moderate duration (45 min to 1 h) for between 10 and 12 weeks’.^37^

The second CPG was issued by the Spanish government and recommended:

‘Studies suggest that group and supervised activities with an aerobic component and of moderate-low intensity (three times a week for at least seven weeks) may be the most appropriate option. However, the relatively small number of existing studies and their limitations should be taken into account. […] the preferences of the patients are essential when recommending physical exercise associated with the treatment plan, and both the possibility of its implementation and the clinical and psychosocial characteristics of the patient should be assessed. […] Therefore, since the evidence regarding the efficacy of physical exercise is limited, the advisability of its recommendation as part of the therapeutic strategy should be assessed individually, taking into account the patient's preferences, and provided that the severity of the picture does not hinder its realization.’^38^

Exercise recommendations were accompanied by sleep and/or nutrition in seven out of eight CPGs. Summary characteristics for included CPGs are outlined in Table 3; details of the CPG recommendations can be found in Supplementary Table 3.

**Table 3:** Characteristics of included CPGs with lifestyle recommendations in ranking order of AGREE-II score for the three key domains

Quality rating for three key domains	Author, year	Country	Disorder	Age group	Lifestyle recommendation					
					Qualitative recommendation			Quantitative recommendation	Level of detail	Evidence
					Nutrition	Sleep	Exercise	Exercise		
High	Grupo de Trabajo, 2018^38^	Spain	Major depression	Children (age 5–11 years) and adolescents (age 12–18 years)	✓	✓	✓	Three times per week for at least 7 weeks	Detailed statements for exercise. Others are a brief statement	Literature review with citations
High	NICE, 2019^37^	UK	Depression	Children and young people (age 5–18 years)	✓	✓	✓	45 min three times per week for 10–12 weeks	Detailed statements for exercise. Others are a brief statement	Literature review with citations
High	MacQueen et al, 2016^41^	Canada	Major depressive disorder	Youth, women and the elderly	✓	✓	✓	–	Brief statement	Literature review with citations
High	Beyond Blue, 2011^42^	Australia	Depression	Adolescents and young adults (age 13–24 years)	✓	✓	✓	–	Brief statement	Literature review with citations
High	Cheung et al, 2018^43^	USA	Depression	Youth (age 10–21 years)	✓	✓	✓	–	Brief statement	None reported
Minimum	Driot et al, 2020^44^	France	Depression	Children and adolescents (age 6–18 years)	✓	✓	✓	–	Brief statement	None reported
Minimum	Seo et al, 2018^45^	Korea	Depressive disorders	Children, adolescents, women and the elderly	✓	^a^	–	–	Brief statement	None reported
Minimum	Ministerio de Salud, 2013^48^	Chile	Depression	Adolescents (age 10–14 years)	✓	✓	✓	–	Brief statement	None reported
Minimum	Garland et al, 2016^47^	Canada	Major depressive disorder	Children and adolescents	–	✓	–	–	Brief statement	None reported
Minimum	Ministerio de Salud, 2013^46^	Chile	Depression	Persons aged ≥15 years	–	–	✓	–	Brief statement	Literature review with citations

✓indicates that it supports the lifestyle recommendation; − indicates that it is not mentioned in the CPG. CPG, clinical practice guideline; AGREE-II, Appraisal of Guidelines for Research and Evaluation, Second Edition.

a. Sleep deprivation.

## Discussion

This umbrella review is the first to summarise clinical practice recommendations for nutrition, sleep and exercise in the treatment of child and youth depression. We identified ten child and/or adolescent CPGs for depression that met the minimum AGREE-II quality criteria and contained recommendations on nutrition, sleep and/or exercise. Six CPGs included relevant recommendations, but did not meet quality criteria for inclusion in this umbrella review. Thirteen CPGs were disregarded because they did not make any mention of sleep, nutrition or exercise. Recommendations for nutrition, sleep and exercise provided by the included CPGs were predominately qualitative, stating the benefits of a balanced diet, healthy sleep and exercise. Quantitative details (e.g. exercise routine) were only outlined in two CPGs (Table 2). Half of the recommendations failed to reference or discuss supporting evidence from the literature.

Vague recommendations place the onus of interpretation for individual treatment plans on clinicians, who may have limited training or expertise regarding the specific linkages between nutrition, sleep or exercise, and youth depression. Additional research and more specific recommendations are needed to help clinicians decide when, for whom and how lifestyle interventions are likely to be most effective, and whether they should be provided as adjunctive treatments alongside psychotherapy or medication, or as interventions in their own right.

Although it might be argued that recommending a healthy lifestyle can do no harm, the possibility of harm arising from these interventions (beyond physical injury during exercise) was not explicitly appraised or discussed in the reviewed CPGs. When implementing stepped-care models, clinicians may decide to follow the broadly phrased lifestyle recommendations currently provided by CPGs, or prefer that youth try to increase exercise, modify their sleep routines or attempt to establish healthier eating patterns before prescribing higher-intensity treatments, such as supportive counselling, psychotherapy or medication. However, if lifestyle interventions were ineffective in alleviating depressive symptoms, prescribing them might cause unnecessary delays in providing proven evidence-based treatments.

Adverse effects may also arise where youth perceive recommendations to make lifestyle changes as dismissive of the severity of their distress or impairment, or of a perceived need for more intensive support. It is also possible that prescribing a healthier lifestyle reinforces self-blame, where youth gain the impression that a personal failure to live a healthier lifestyle has caused their depression. Lifestyle prescriptions may also fuel arguments between parents and youth, where parents have already attempted to encourage more healthy eating, sleep and activity patterns. Similarly, severe depressive symptoms may interfere with a young person's ability to implement lifestyle advice, which may reinforce feelings of guilt and failure. Additionally, sedentary behaviour among parents may model sedentary lifestyle habits as normal, making such interventions more difficult to implement. For example, parents who are sedentary owing to health reasons, work or family commitments may not have time for exercise, and may inadvertently be modelling sedentary behaviour as normal.

A lack of youth and/or family stakeholder involvement in the development of the CPGs (except for the NICE CPG) was one factor that contributed to the downgrading of AGREE-II scores. Future CPGs should seek to incorporate the perspective of youth, and appraise the cognitive and emotional effects that broadly phrased lifestyle recommendations might have when implemented in primary or routine specialist care.

### Strengths and limitations

The strengths of this umbrella review relate to the systematic methodology applied herein, which included strict inclusion/exclusion criteria, duplicate screening, an AGREE-II quality assessment and the exclusion of CPGs that did not meet minimum quality cut-offs. Although not exhaustive, the inclusion of some non-English CPGs enabled the consideration of a more global sample of recommendations.

Some limitations should be considered when interpreting the study findings. One is the limited capacity within the study for the duplicate quality assessment of French-, German- and Spanish-language CPGs, as well as the capacity to screen and include CPGs in other languages. Additionally, all the nutrition and sleep recommendations were brief statements and half of the included CPGs lacked citations to support the lifestyle recommendations made, making it impossible to review the quality of evidence underlying them. Of the 13 candidate paediatric depression CPGs (Supplementary Fig. 1) that were excluded because of a lack of recommendations regarding nutrition, sleep or exercise, it remains unclear whether they explicitly chose not to mention lifestyle recommendations because of the limited evidence base, or whether these lifestyle factors were not considered when reviewing the evidence for different treatment options. Other limitations relate to the body of primary evidence supporting lifestyle recommendations. There is a considerable epidemiological evidence base examining the impact of lifestyle factors, such as nutrition,^15,17^ sleep^21,22^ and exercise,^27,39^ on the development and maintenance of child and youth depression. However, there is limited evidence on the efficacy and effectiveness of these lifestyle factors as treatment ingredients, which is reflected by a lack of detail in the recommendations.

As knowledge about the efficacy surrounding lifestyle interventions grows, the findings may support prescribing lifestyle changes to youth that are waitlisted to receive evidence-based treatments. As such, lifestyle interventions could be part of a wider catalogue of intervention strategies that youth can use without being directly accompanied by a health professional.^40^ Further research is needed to determine whether lifestyle interventions should precede more intensive interventions, whether they should be provided alongside other psychosocial interventions or medication, or whether they are most helpful when provided to youth who do not respond to evidence-based treatments. Multi-centre trials engaging youth across different treatment sites may be one avenue for increasing statistical power and, potentially, the representativeness of study samples to support robust conclusions for lifestyle interventions. Future research should also address the risks and benefits of each approach, including the use of lifestyle interventions in stepped-care models and primary care.

In conclusion, there is some evidence that lifestyle interventions can have beneficial effects on depression, but research is still limited and there are very few well-designed, randomised controlled trials. Traditionally, interventions related to nutrition, sleep and exercise have not been included in the mental health toolbox. This umbrella review found that ten CPGs for paediatric depression with minimum or high quality contained mainly brief and non-specific statements concerning nutrition, sleep or exercise, that left room for multiple interpretations; for example, concrete treatment plans or dosage suggestions were rarely provided. Half of the included CPGs did not back their recommendations with references to the existing literature. Although the evidence base is growing, lifestyle recommendations are hampered by inadequate study designs and a field of study in the early stages.

Future research should build on associations between nutrition, sleep or exercise and depressive symptoms. High-quality, multi-centred trials are needed that examine the effectiveness of lifestyle interventions as a precursor to higher-intensity treatments, as a concurrent treatment or as an option for treatment-resistant depression. Similarly, trials are needed that assess the benefits of using these interventions in different care settings (e.g. primary care, specialist services), and across different socioeconomic and ethnic groups. Future studies should examine whether lifestyle interventions can have any adverse effects on young people's treatment motivation and well-being. It is important to include youth with lived experience of depression and their families in the design and interpretation of future studies, as well as in the formulation of future CPGs, to ensure relevant interventions are acceptable and meaningful to youth.

This review suggests that lifestyle interventions as ingredients for treatment in child and youth depression hold promise and have been addressed in several high-quality CPGs. However, the current body of evidence needed to develop strong CPG recommendations is scant. Progress calls for further rigorous trials to fill key knowledge gaps regarding the role of these factors alone, and in combination with psychotherapy and medication.

## Data Availability

Data availability is not applicable to this article as no new data were created or analysed in this study.

## Supplementary material

For supplementary material accompanying this paper visit http://dx.doi.org/10.1192/bjo.2021.1020. click here to view supplementary material
