# Supplementary material for: Eating, sleeping and moving recommendations in clinical practice guidelines for paediatric depression: umbrella review
Source: BJPsych Open. 2021 Oct 5;7(6):e185. doi: 10.1192/bjo.2021.1020 (PMC8503910; doi:10.1192/bjo.2021.1020)
Supplement: Supplementary file 1 [file S2056472421010206sup001.docx]

**Supplementary Materials**

**Supplementary Table 1**. Search Strategy for Pediatric Clinical Practice Guidelines on Depression

| ***Medline*** Database: Ovid MEDLINE: Epub Ahead of Print, In-Process & Other Non-Indexed Citations, Ovid MEDLINE® Daily and Ovid MEDLINE® <1946-Present> | |
| --- | --- |
| 1 | exp Depression/ |
| 2 | exp Depressive Disorder/ |
| 3 | depress$.mp. |
| 4 | dysthymic$.mp. |
| 5 | dysthymia$.mp. |
| 6 | exp clinical pathway/ |
| 7 | exp clinical protocol/ |
| 8 | exp consensus/ |
| 9 | exp consensus development conference/ |
| 10 | exp consensus development conferences as topic/ |
| 11 | critical pathways/ |
| 12 | exp guideline/ |
| 13 | guidelines as topic/ |
| 14 | exp practice guideline/ |
| 15 | practice guidelines as topic/ |
| 16 | health planning guidelines/ |
| 17 | treatment guideline$.mp. |
| 18 | guideline or practice guideline or consensus development conference or consensus development.pt. |
| 19 | position statement* or policy statement* or practice parameter* or best practice*).mp. |
| 20 | standard$ or guideline$).ti. |
| 21 | ((practice$ or practise$ or treatment*) adj3 guideline*).ab. |
| 22 | ((practice or treatment*) adj guideline*).mp. |
| 23 | (CPG or CPGs).mp. |
| 24 | Consensus*.mp. |
| 25 | ((critical or clinical or practice) adj2 (path or paths or pathway or pathways or protocol*)).mp. |
| 26 | recommendat*.mp. |
| 27 | (care adj2 (standard or path or paths or pathway or pathways or map or maps or plan or plans)).mp. |
| 28 | (algorithm* adj2 (screen$ or examination$ or test or tested or testing or assess* or diagnosis or diagnoses or diagnosed or diagnosing)).mp. |
| 29 | (algorithm* adj2 (1hemotherapy1py* or psychopharm$ 1hemotherapy* or chemotreatment* or therap* or treat* or intervent*)).mp. |
| 30 | exp child/ |
| 31 | exp adolescents/ |
| 32 | exp Young Adult/ |
| 33 | (child* or juvenile$ or pubescen* or teen* or preteen* or tween* or youth$ or adoles$ or young$ adult$ or emerg$ adult$).mp. |
| 34 | (child* or juvenile$ or pubescen* or teen* or preteen* or tween* or youth$ or adoles$ or young$ adult$ or emerg$ adult$ or pediatric* or paediatric$).mp. |
| 35 | (child* or juvenile$ or pubescen* or teen* or preteen* or tween* or youth$ or adoles$ or young$ adult$ or emerg$ adult$ or pediatric* or paediatric$).jw. |
| 36 | or/1-5 [depression set] |
| 37 | or/5-28 [guideline set] |
| 38 | or/33-35 [child set] |
| 39 | 36 and 37 and 38 |
| 40 | limit 39 to yr=”2017 -Current” |
| 41 | limit 40 to (english or french or german or spanish) |
| 42 | limit 39 to yr=”2005 -2016” |
| 43 | limit 42 to (french or german or spanish) |

**Supplementary Table 2**. AGREE-II Scores for CPGs evaluated for quality (n=16)

| Guideline | AGREE-II Domain | | | | | | | Quality Rating | |
| --- | --- | --- | --- | --- | --- | --- | --- | --- | --- |
| Author, Year | Combined Overall Rating | Scope & Purpose | Stakeholder Involvement | Rigor of Development | Clarity of Presentation | Applicability | Editorial Independence | 3 key scores | 3 non-key scores |
| Beyond Blue, 2011 | 83 | 97 | 75 | 82 | 92 | 42 | 71 | 76 | 77 |
| Birmaher et al., 2007 | 50 | 61 | 31 | 48 | 50 | 27 | 50 | 43 | 46 |
| Cheung et al., 2018 | 83 | 69 | 81 | 63 | 92 | 52 | 83 | 76 | 71 |
| Dolle et al., 2014 | 33 | 56 | 33 | 56 | 56 | 8 | 33 | 49 | 32 |
| Driot et al., 2020 | 50 | 67 | 83 | 44 | 44 | 29 | 75 | 67 | 47 |
| Garland et al., 2016 | 58 | 78 | 53 | 63 | 56 | 48 | 42 | 53 | 61 |
| Grupo de Trabajo, 2018 | 83 | 100 | 83 | 71 | 89 | 58 | 100 | 85 | 82 |
| Guidelines and Protocols Advisory Committee, 2010 | 42 | 56 | 36 | 36 | 64 | 29 | 33 | 35 | 50 |
| Haute Autorité de Santé, 2014 | 67 | 78 | 44 | 54 | 89 | 38 | 50 | 49 | 68 |
| Kaiser Permante, 2018 | 42 | 72 | 39 | 52 | 72 | 44 | 46 | 46 | 63 |
| MacQueen et al., 2016] | 75 | 89 | 78 | 70 | 67 | 60 | 83 | 77 | 72 |
| Ministerio de Salud, 2013 | 67 | 83 | 61 | 67 | 67 | 29 | 25 | 51 | 60 |
| Ministerio de Salud, 2014 | 67 | 89 | 61 | 58 | 72 | 29 | 42 | 54 | 63 |
| NICE, 2019 | 92 | 83 | 72 | 72 | 83 | 83 | 92 | 79 | 83 |
| Orygen et al., 2017 | 50 | 86 | 39 | 39 | 69 | 38 | 33 | 37 | 64 |
| Seo et al., 2018 | 67 | 75 | 64 | 50 | 78 | 56 | 71 | 62 | 70 |

^a^ Key Domains (Stakeholder Involvement, Rigor of Development, Editorial Independence); ^b^ Non-Key Domains (Scope & Purpose, Clarity of Presentation, Applicability)

**Supplementary Table 3. Recommendations of CPGs evaluated for quality (n=16)**

| Author, Year | Country | Language | Disorder | Age-group | CPG Lifestyle Recommendation Details |
| --- | --- | --- | --- | --- | --- |
| Beyond Blue, 2011 | Australia | English | Depression | Adolescents and young adults (13-24 years) | - “Relapse prevention strategies for young people: Follow a healthy lifestyle, including exercise, proper nutrition and good sleep habits” - “There is limited evidence suggesting that moderate exercise levels and high exercise levels had a similar impact on depressive symptoms in adolescents at risk of depression”. - “Very limited evidence suggests that regular group exercise (e.g. jogging or yoga) is an effective means of reducing depressive symptoms in the short term in young adults with depressive symptoms, although there is no evidence of its effectiveness in the long term or its ability to prevent the transition to depression.” - “[...] while many individuals note that their sense of wellbeing is enhanced by physical activity, based on current evidence, physical activity cannot be recommended as an effective treatment for depression in young people” - “Initial approaches for managing depressive symptoms: There is good evidence that relaxation, physical activity, and healthy sleep patterns promote feelings of wellbeing.” - Appendix 6 lists Australian guidelines for diet and physical activity |
| Birmaher et al., 2007 | United States | English | Depressive Disorders | Children and adolescents | - “The strategies for the prevention of onset or of recurrence of depression should include the amelioration of risk factors associated with this disorder. In addition, prevention may also include lifestyle modifications: regular and adequate sleep, exercise, a coping plan for stress (e.g., meditation, yoga, exercise, social activities)” |
| Cheung et al., 2018 | United States | English | Depression | Youth (10-21 years) | - “Common sense approaches such as the prescription of physical exercise, sleep hygiene, and adequate nutrition should also be used in the management of these patients.” |
| Dolle et al., 2014 | Germany | German | Depression | Children & adolescents (3-18 years) | - At the time of writing, the CPG reported that the evidence reviewed did not support any recommendation for sleep, omega-3 fatty acid or exercise |
| Driot et al., 2020 | France | French | Depression | Children and adolescents (6-18 years) | - “Management algorithm [...] practical elements of management: [...] life-hygiene: balanced diet, sufficient sleep, and regular physical activity.” |
| Garland et al., 2016 | Canada | English | Major Depressive Disorder | child and adolescent | - “When faced with a child or adolescent with mild depression or anxiety symptoms the most appropriate initial step should be supportive treatment including psychoeducation, sleep hygiene” |
| Grupo de trabajo de la actualización, 2018 | Spain | Spanish | Major Depression | Children (5-11 years) and adolescents (12-18 years) | - Recommend healthy lifestyle habits including balanced nutrition, sleep pattern and physical exercise - “Studies suggest that group and supervised activities with an aerobic component and of moderate-low intensity (3 times a week for at least 7 weeks) may be the most appropriate option. However, the relatively small number of existing studies and their limitations should be taken into account. [...]Therefore, since the evidence regarding the efficacy of physical exercise is limited, the advisability of its recommendation as part of the therapeutic strategy should be assessed individually, taking into account the patient's preferences, and provided that the severity of the picture does not hinder its realization." - “Sleep problems can be part of depression. Major sleep problems may require medication.” - “In major depression in childhood and adolescence, the use of light therapy, polyunsaturated omega-3 fatty acids, glutamine, St. John's wort (Hypericum perforatum), S-adenosyl methionine, vitamin C or tryptophan is not recommended due to the lack of evidence on its effectiveness.” - “For parents: Make sure [he/she] gets a good diet and is exercising regularly. Example: drink enough water, eat vegetables and fruits, go for a walk once a day with him, take a bike ride” |
| Guidelines and Protocols Advisory Committee, 2010 | Canada | English | Anxiety & Depression | Children & adolescents (≤18 years) | - “[for parents] Remember the basics: regular sleep, eating and exercise routines […]” |
| Haute Autorité de Santé (HAS), 2014 | France | French | Depression | Adolescents (12-18 years) | - "Supportive therapy should include: […] psychoeducation: promotion of self-care, hygiene-dietetic rules (physical activity, sleep hygiene and diet), [...]” |
| Kaiser Permanente, 2018 | United States | English | Depression | Adolescents (12-17 years) and adults | - “Providers can engage in behavioral activation by encouraging patients to consider […] engaging in physical activity” - “For patients who are reluctant to take traditional antidepressants, use of omega-3 fatty acids as a standalone medication could be considered. There is some evidence that omega-3 fatty acids are more effective than placebo in reducing depression symptoms, but not enough to recommend them over standard antidepressants. Use of omega-3s as an adjunct medication is a reasonable option. For patients who are reluctant to take traditional antidepressants, use of omega-3 fatty acids as a standalone medication could be considered. Omega-3s are available as an over the counter supplement in fish oil. Most fish oil supplements contain a combination of EPA (eicosapentaenoic acid) and DHA (docosahexaenoic acid). Data suggests that EPA predominant formulations demonstrate efficacy in the treatment of depression. The recommended dose of EPA is 1–2 g daily, divided into morning and evening doses. With evidence findings similar to those on omega-3s, S-adenosyl-L-methionine (SAMe) may be effective as an adjunct to traditional antidepressants and may help treatment-resistant patients achieve remission of depression. The recommended dose of SAMe is 400–800 mg daily, divided into morning and evening doses. Overall, there is evidence to support the use of omega-3 fatty acids and SAMe as monotherapy or adjunct treatment to reduce depressive symptoms in the short term. However, the strength of evidence is low.” |
| MacQueen et al., 2016 | Canada | English | Major Depressive Disorder | Youth, Women, and the Elderly | - “Supportive approaches include psychoeducation, active and empathetic listening, and lifestyle advice, including the benefits of good sleep hygiene, proper eating habits, and exercise.” |
| Ministerio de Salud, 2013 | Chile | Spanish | Depression | Persons ≥15 years | - "Indicate a structured program of physical activity for the treatment of people with depression." |
| Ministerio de Salud, 2014 | Chile | Spanish | Depression | Adolescents (10-14 years) | - “The promotion of healthy lifestyles, such as regular physical activity, healthy eating and adequate sleep schedules, is recommended as part of therapeutic intervention.” |
| NICE, 2019 | United Kingdom | English | Depression | Children and young people (5-18 years) | - “Treatment and considerations in all settings:   - A child or young person with depression should be offered advice about sleep hygiene and anxiety management.”   - “A child or young person with depression should be offered advice about nutrition and the benefits of a balanced diet.”   - “A child or young person with depression should be offered advice on the benefits of regular exercise and encouraged to consider following a structured and supervised exercise program of typically up to 3 sessions per week of moderate duration (45 minutes to 1 hour) for between 10 and 12 weeks. (page 10) Brief psychosocial intervention (BPI) sleep, diet and physical activity.” |
| Orygen et al., 2017 | Australia | English | Depression | Adolescents (12-18 years) and young adults (18-25 years) | - “psychoeducation information and encouragement on sleep hygiene, maintaining a healthy diet and the benefits of regular exercise.” - “Exercising as an important adjunct to treatment information (particularly about depression, psychoeducation about the role of exercise, diet, and sleep in reducing depressive symptoms)” |
| Seo et al., 2018 | Korea | English | Depressive Disorders | Children and adolescents; elderly, Women | - “Light therapy, nutritional therapy (omega-3, megavitamin), vagus nerve stimulation, S-adenosylmethionine, deep brain stimulation, and sleep deprivation were considered as second-line treatment options for MDD. These are recommended as "Choice of complementary or novel agents for treatment-resistant depressive disorder " |
